# The potential interaction between medical treatment and radioiodine treatment success: A systematic review

**DOI:** 10.3389/fendo.2022.1061555

**Published:** 2023-01-04

**Authors:** Riazul Zannat, Jonathan Lee, Jameel Muzaffar, Martin L. Read, Katie Brookes, Neil Sharma, Kristien Boelaert, Christopher J. McCabe, Hannah R. Nieto

**Affiliations:** ^1^ Institute of Metabolism and Systems Research, University of Birmingham, Birmingham, United Kingdom; ^2^ Department of Ear, Nose and Throat Surgery, Warwick Hospital, University Hospitals of South Warwickshire NHS Foundation Trust, Birmingham, United Kingdom; ^3^ Department of Clinical Neurosciences, University of Cambridge, Cambridge, United Kingdom; ^4^ Department of Ear, Nose and Throat Surgery, University Hospitals Birmingham NHS Foundation Trust, Birmingham, United Kingdom; ^5^ Institute of Cancer and Genomic Sciences, University of Birmingham, Birmingham, United Kingdom; ^6^ Institute of Applied Health Research, University of Birmingham, Birmingham, United Kingdom; ^7^ Birmingham Health Partners, University of Birmingham, Birmingham, United Kingdom

**Keywords:** radioiodine, thyroid cancer, hyperthyroidism, prednisone, glycididazole sodium, lithium, carbimazole, antithyroid drugs

## Abstract

**Introduction:**

Radioactive iodine (RAI) therapy is a critical component in the post-surgical management of thyroid cancer patients, as well as being a central therapeutic option in the treatment of hyperthyroidism. Previous work suggests that antithyroid drugs hinder the efficacy of RAI therapy in patients. However, the effects of other background medications on RAI treatment efficacy have not been evaluated. Therefore, we performed a systematic review and meta-analysis investigating the potential off-target effects of medication on RAI therapy in patients with thyroid cancer and hyperthyroidism.

**Methods:**

Systematic review and meta-analysis according to the 2020 PRISMA guidelines. Databases searched: MEDLINE, EMBASE and Cochrane Library for studies published between 2001 and 2021.

**Results:**

Sixty-nine unique studies were identified. After screening, 17 studies with 3313 participants were included. One study investigated thyroid cancer, with the rest targeted to hyperthyroidism. The majority of studies evaluated the effects of antithyroid drugs; the other drugs studied included lithium, prednisone and glycididazole sodium. Antithyroid drugs were associated with negative impacts on post-RAI outcomes (n = 5 studies, RR = 0.81, p = 0.02). However, meta-analysis found moderate heterogeneity between studies (I2 = 51%, τ2 = 0.0199, p = 0.08). Interestingly, lithium (n = 3 studies), prednisone (n = 1 study) and glycididazole (n = 1 study) appeared to have positive impacts on post-RAI outcomes upon qualitative analysis.

**Conclusion:**

Our systematic review strengthens previous work on antithyroid medication effects on RAI, and highlights that this field remains under researched especially for background medications unrelated to thyroid disease, with very few papers on non-thyroid medications published.

**Systematic review registration:**

https://www.crd.york.ac.uk/prospero/display_record.php, identifier CRD42021274026.

## Introduction

### Rationale

β-emitting radioiodide (^131^I) has been utilized for over 75 years to safely and efficiently destroy remaining thyroid cancer cells post-surgery, and to ablate thyroid cells in patients with hyperthyroidism (1). The sodium iodide symporter (NIS) is the sole known transporter of iodide into human cells (2). Different patient and disease characteristics influence treatment course and outcome, but a fuller understanding of the factors which impact treatment success might facilitate enhanced outcomes in the management of both thyroid tumors and hyperthyroidism.

The ability of NIS to uptake radioiodide is diminished in 25-50% of thyroid cancer patients, due to reduced expression and mislocalization away from the plasma membrane (PM) (3, 4), its only site of transport activity. The regulation of NIS expression and function is complex, being governed *via* transcription factors, miRNAs, promoter methylation and histone acetylation, hormonal signaling, post-translational modification, and by iodide itself (5–10). Pioneering recent studies in differentiated thyroid cancer have shown that treatment with MEK and/or BRAF inhibitors – as well as other pathway targeted approaches - increases ^131^I avidity in subsets of patients, raising the possibility of achieving a tumor response following ^131^I treatment (11–14). However, what is much less well understood is the impact of contemporaneous therapies at the time of treatment on clinical success and outcome.

Clinical predictors of 131I therapy failure in differentiated thyroid cancer may include tumor focality and lymph node invasion at surgical resection (15), although predictors of treatment success in hyperthyroidism are less clear. Walter et al. (16) first highlighted the counterproductive effect of antithyroid drugs on radioactive iodine treatment. In particular, drugs such as carbimazole and propylthiouracil (PTU) had a negative influence on RAI uptake when taken in the week prior to RAI therapy or post-RAI therapy (16). These findings suggest that antithyroid drugs may have influenced RAI kinetics, possibly mediated by a reduction in free radicals (16). Further studies are needed to investigate whether the efficacy of RAI therapy is similarly diminished by off-target effects arising from other coincident medications.

More widely, it has been documented that exposure to background pollutants can significantly modulate thyroid function and treatment. Phthalate exposure in pregnancy (17), for example, has been shown to modulate thyroid function, and perchlorate, a competitive inhibitor of NIS, is detrimental to radioactive iodine treatment (18, 19). As such, patients’ medication (administered for other conditions) might potentially interact with the cellular mechanisms which mediate radioactive iodine uptake, critically affecting outcomes in both hyperthyroidism treatment and thyroid cancer ablation therapy. Our study therefore seeks to better understand possible interactions of common medications on the efficacy of radioactive iodine administration.

### Objectives and research question

The aim of this systematic review is to evaluate whether medications taken by patients with thyroid cancer or hyperthyroidism receiving RAI significantly impact RAI treatment success (i.e. response rate for thyroid cancer or if patients were rendered euthyroid or hypothyroid for hyperthyroidism). This review was prospectively registered on PROSPERO to minimize bias and prevent duplication and was designed according to Preferred Reporting Items for Systematic Reviews and Meta-Analyses (PRISMA) statement (20).

## Methods

### Search strategy and data sources

The search strategy was developed in conjunction with an information specialist librarian (Evidence search: ‘Do commonly used drugs diminish the efficacy of radioiodine treatment in hyperthyroidism and thyroid cancer?’ performed by Jennifer Manders on 18th August, 2021. BIRMINGHAM, UK: University Hospitals Birmingham (UHB) Library and Knowledge Service). Records published between 2001 and 2021 were collected by searching systematically on Medline, EMBASE and the Cochrane Library. Examples of search terms used can be seen in **Table 1** of the current paper and **Table 1** of the **Supplementary Material**. Studies were performed on adult patients only and no other limitations were applied to the search. Following this, 67 duplicated results were removed. Five papers were identified for eligibility screening through citation searching.

**Table 1:** Search strategy on MEDLINE for records published between 2001 and 2021.

Search term	Result
**1. (hyperthyroidism).ti,ab**	20752
**2. (“overactive thyroid”).ti,ab**	21
**3. (Thyroid ADJ1 (Cancer OR neoplasms)).ti,ab**	25726
**4. (“papillary carcinoma”).ti,ab**	5725
**5. (“follicular carcinoma”).ti,ab**	2018
**6. ((Medullary OR anaplastic) ADJ1 “thyroid carcinoma”).ti,ab**	5304
**7. (1 OR 2 OR 3 OR 4 OR 5 OR 6)**	55365
**8. (“radioactive iodine”).ti,ab**	5862
**9. (Radioiodine).ti,ab**	9215
**10. (I-131).ti,ab**	4315
**11. (8 OR 9 OR 10)**	17905
**12. (Amiodarone).ti,ab**	9690
**13. (Alendronate).ti,ab**	4812
**14. (Clindamycin).ti,ab**	11112
**15. (Escitalopram).ti,ab**	2710
**16. (Mebeverine).ti,ab**	165
**17. (“Enalapril maleate”).ti,ab**	397
**18. (Diltiazem).ti,ab**	8680
**19. (Amitriptyline).ti,ab**	158
**20. (Exemestane).ti,ab**	1368
**21. (Raloxifene).ti,ab**	3369
**22. (Spironolactone).ti,ab**	6040
**23. (Alfacalcidol).ti,ab**	645
**24. (Digoxin).ti,ab**	11770
**25. (Celecoxib).ti,ab**	6552
**26. (“Cyproterone acetate”).ti,ab**	2503
**27. (12 OR 13 OR 14 OR 15 OR 16 OR 17 OR 18 OR 19 OR 20 OR 21 OR 22 OR 23 OR 24 OR 25 OR 26)**	68097
**28. (Medication).ti,ab**	231230
**29. (Medicine).ti,ab**	488876
**30. (Drug).ti,ab**	1227922
**31. (27 OR 28 OR 29 OR 30)**	1908684
**32. (7 AND 11 AND 31)**	828
**33. 32 [DT 2001-2021]**	454

In addition to using generic search terms to capture all medications, specific drugs from a previous screening study (21) were included in the search. ti, title; ab, abstract.

### Study selection and data extraction

Records were screened for inclusion according to the inclusion and exclusion criteria summarized in **Table 2**. Randomized-control trials (RCTs), case-control studies or cohort studies that studied the effect of medication on radioactive iodine treatment prognosis in human patients with thyroid cancer or hyperthyroidism were included, whilst systematic reviews and laboratory investigations were excluded.

**Table 2:** Inclusion and exclusion criteria.

Inclusion criteria	Exclusion criteria
**Adults with hyperthyroidism or thyroid cancer**	Children, animals
**Randomised control trials, case-control studies, cohort studies**	Review, systematic review, laboratory research
**Medication having an effect on RAI treatment**	Non-English language

Initially, the 69 studies gathered from the search were screened by title and abstract by two independent reviewers [RZ and JL]. Disagreements were resolved by discussion and a third reviewer [HN]. Following the initial screening, the full-text articles of 49 studies were obtained for a full-text screening against the aforementioned inclusion criteria. The reference lists of these papers were searched, resulting in the screening of an additional five articles. In total, 17 articles were included for data extraction.

Data were extracted and recorded in a standard Excel spreadsheet by one reviewer [RZ] and checked by a separate reviewer [JL]. Patient characteristics, study characteristics and patient outcomes were recorded.

### Quality assessment

To assess the risk of bias of the randomized-control trials, the Cochrane risk-of-bias tool for randomized trials (RoB 2) was used (22). This tool allowed for the appraisal of the randomization process, deviations, measurement and reporting of outcomes of each randomized control trial. To assess the risk of bias of the case-control studies and cohort studies, the Critical Appraisal Skills Programme (CASP) checklists for case-control studies and cohort studies were used (23, 24). These checklists evaluate the validity of the study, the method, the results and their relevance for our study population.

### Data synthesis and analysis

A meta-analysis was performed examining cure rates after radioactive iodine for patients on carbimazole or methimazole vs control (R version 4.1.2, metabin package). This was conducted as a random effects meta-analysis, with study heterogeneity examined using the I^2^ statistic. This was considered low at < 50%, moderate at 50 to 75% and high at >75%. The weight to each study, relative risk (RR) and 95% confidence intervals were calculated and τ^2^ using the Paule-Mandel estimator. A subset analysis of randomized vs non-randomized studies was also performed.

## Results

### Description of studies

**Figure 1:**
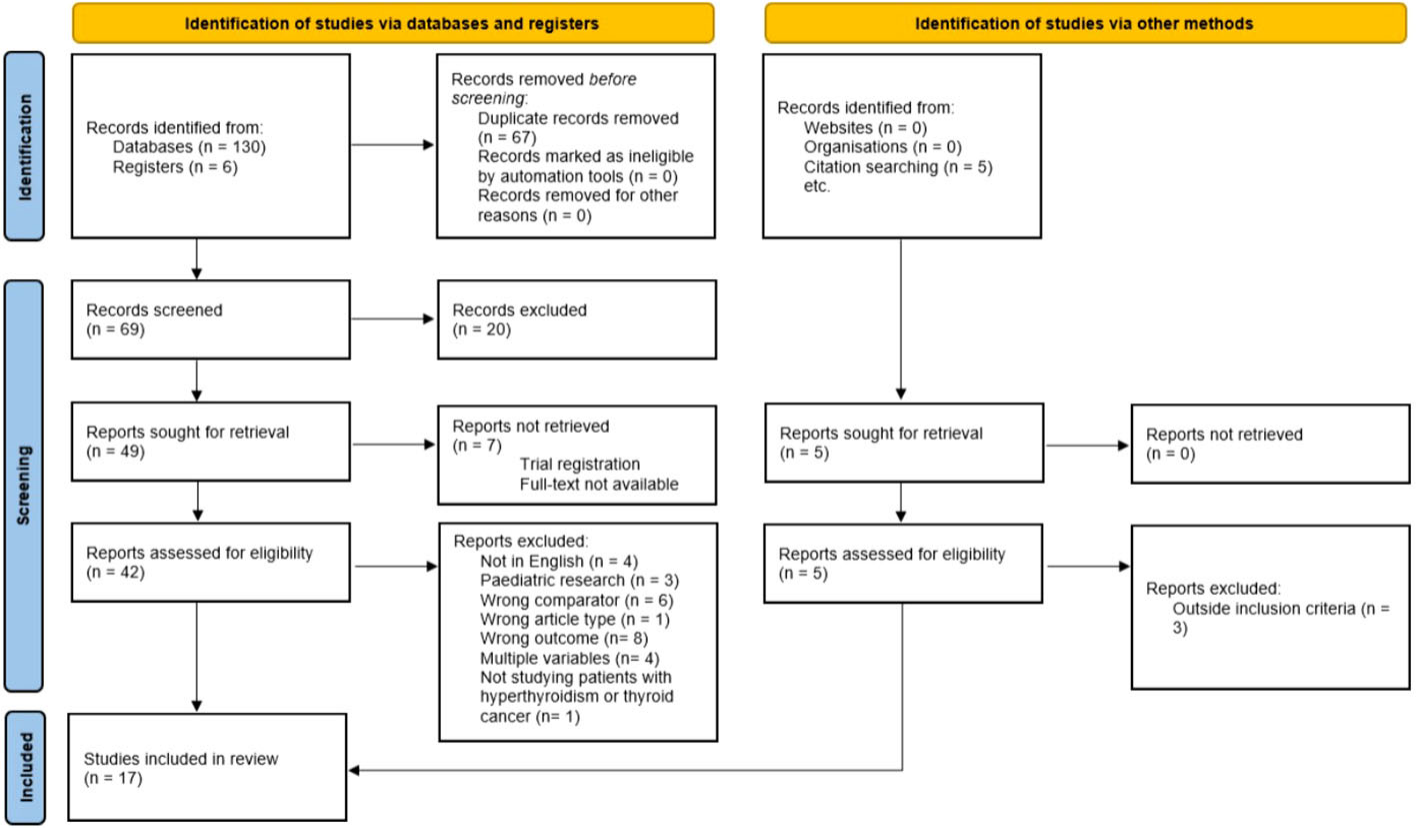
PRISMA 2020 flow diagram for systematic review. From: Page MJ, McKenzie JE, Bossuyt PM, Boutron I, Hoffmann TC, Mulrow CD, et al. The PRISMA 2020 statement: an updated guideline for reporting systematic reviews. BMJ 2021;372:n71. doi: 10.1136/bmj.n71. For more information, visit: http://www.prisma-statement.org/.

The initial search found 136 studies from which 67 duplicate studies were removed. Following this, 69 studies were screened by title and abstract against the inclusion and exclusion criteria (**Table 2**) and 20 studies were excluded. Seven papers could not be retrieved for full-text screening. Forty-two studies were subject to full-text screening and a further 27 were excluded (**Figure 1**). An additional five studies were found by citation searching and two of these were eligible for full-text screening. **Figure 1** shows the PRISMA flow diagram of the selection process.

A total of 17 studies were included in this systematic review with 3313 patients. Of the 17 included studies, nine were RCTs, seven were cohort studies and one was a case-control study. All studies investigated outcomes in patients with hyperthyroidism with the exception of Wen et al. (25) who investigated outcomes in patients with thyroid cancer.

In every study there were more female participants than male participants, with mean ages ranging from 36.2 years to 65.3 years. Most studies investigated the effect of antithyroid drugs, such as methimazole, thiamazole, carbimazole and propylthiouracil (26–37) on RAI outcomes. Three studies researched lithium (38–40), one study researched lithium carbonate and prednisone in addition to thiamazole (34), and one study researched glycididazole sodium (25).

Thyroid status was defined as euthyroid, hypothyroid or hyperthyroid and was measured using standard diagnostic assays. Six studies (27, 29, 30, 34, 35, 40) evaluated thyroid status using free triiodothyronine (FT3), free thyroxine (FT4) and thyroid stimulating hormone (TSH) concentrations. Studies used differing thyroid blood assays and the need for repeat RAI doses to assess thyroid status (26, 28, 31, 32, 36, 37). The outcomes for patients with thyroid cancer were measured by evaluating unstimulated thyroglobulin (Tg) levels and ^131^I whole body scanning at 12 and 24 weeks following RAI administration in the study by Wen et al. (25). Four studies (33, 38, 39, 41) did not provide details of how thyroid status was assessed. A summary of study characteristics is provided in **Table 3**.

**Table 3:** Study characteristics.

Author	Year	Country	Number of patients	Indication for RAI	Study design	Medication	Comparator	RAI dose
**Andrade et al.** (26)	2001	Brazil	61	Hyperthyroidism	RCT	Methimazole	No methimazole	Uptake-adapted
**Bal et al.** (38)	2002	India	316	Hyperthyroidism	RCT	Lithium	No lithium	218- 433 MBq
**Bogazzi et al.** (39)	2002	Italy	36	Hyperthyroidism	RCT	Lithium	No lithium and different durations and starting points for lithium treatment	479- 550 MBq
**Bogazzi et al.** (40)	2010	Italy	647	Hyperthyroidism	Cohort	Lithium	No lithium	Uptake-adapted
**Bonnema et al.** (27)	2006	Denmark	75	Hyperthyroidism	RCT	Methimazole	Discontinuation of methimazole 8 days before RAI therapy	Uptake-adapted
**Bonnema et al.** (28)	2011	Denmark	100	Hyperthyroidism	RCT	Methimazole+levothyroxine	Discontinuation of methimazole 8 days before RAI therapy	Uptake-adapted
**Braga et al.** (29)	2002	America	34	Hyperthyroidism	RCT	Methimazole	No drug	Uptake-adapted
**Eschmann et al.** (30)	2006	Germany	141	Hyperthyroidism	Cohort	Carbimazole, methimazole, propylthiouracil	Continuation of ATD after RAI therapy	Uptake-adapted
**Karyampudi et al.** (35)	2014	India	70	Hyperthyroidism	Cohort	Carbimazole	No ATD	5 mCi
**Körber et al.** (31)	2001	Germany	707	Hyperthyroidism	Cohort	Carbimazole, methimazole	Discontinuation of ATD at least 14 days before RAI	Uptake-adapted
**Oszukowska et al.** (34)	2010	Poland	200	Hyperthyroidism	Case-control	Thiamazole, lithium carbonate, prednisone	Drugs compared, no placebo	Uptake-adapted
**Płazińska et al.** (41)	2011	Poland	256	Hyperthyroidism	RCT	Lithium carbonate	No lithium	Uptake-adapted
**Santos et al.** (32)	2004	Brazil	100	Hyperthyroidism	Cohort	Propylthiouracil, methimazole	No drug	10 mCi
**Walter et al.** (36)	2006	Switzerland	207	Hyperthyroidism	RCT	Carbimazole	No carbimazole or carbimazole discontinuation 3 days prior to RAI therapy	Uptake-adapted
**Walter et al.** (37)	2009	Switzerland	228	Hyperthyroidism	Cohort	Carbimazole	No carbimazole	Uptake-adapted
**Wen et al.** (25)	2015	China	53	Thyroid cancer	RCT	Glycididazole sodium	Saline (monotherapy)	4.44 GBq
**Zantut-Wittman et al.** (33)	2005	Brazil	82	Hyperthyroidism	Cohort	Methimazole, propylthiouracil	Withdrawal of ATD 2-30 days before RAI therapy	370 MBq

In most of the studies of patients with hyperthyroidism (26–40), success of RAI treatment was defined as either euthyroidism or hypothyroidism. In the one study of patients with thyroid cancer (25), RAI treatment success was defined as the absence of thyroid bed uptake ^131^I and Tg concentrations below 10 μg/ml with no serum Tg antibodies.

### Quality of studies

The majority of the studies were prospective, with three being retrospective. The study sample sizes were generally small, and ranged from 53 to 707 patients in the final analysis.

A summary of the Cochrane risk of bias assessment is provided in **Table 4**. The overall quality of the nine RCTs (25–29, 36, 38, 39, 41) was poor with five reaching high concern and three with some concerns. Six studies were assessed to be of some concern in domain 1 due to lack of information regarding concealment of randomization (**Table 4**). All studies except for two (29, 38) were assessed to be of high or some concern in regards to domain 2 due to the lack of mention of placebo use in the control groups or uneven or large losses to follow-up that were not accounted for in the analysis. Six of the nine studies were assessed to be of low concern in domain 3, the three studies that were of high concern or some concern had missing outcome data. Two studies showed differences in the measurement of outcomes between intervention and control groups, thus were assessed as being of high concern in domain 4. Four studies were assessed to be of high concern in domain 5 due to the absence of post-RAI outcome results or because the method of outcome measurement i.e. thyroid status post-RAI was not mentioned as part of the method.

**Table 4:** Cochrane risk-of-bias tool for randomized trials (RoB 2).

Authors	Domain 1	Domain 2	Domain 3	Domain 4	Domain 5	Overall
Andrade et al, 2001 (26)						
Bal et al, 2002 (38)						
Bogazzi et al, 2002 (39)						
Bonnema et al, 2006 (27)						
Bonnema et al, 2011 (28)						
Braga et al, 2002 (29)						
Płazińska et al, 2011 (41)						
Walter et al, 2006 (36)						
Wen et al, 2015 (25)						

Red indicates high concerns, yellow indicates some concerns and green indicates low concerns. Domain 1: Risk of bias arising from the randomization process. Domain 2: Risk of bias due to deviations from the intended interventions (effect of assignment to intervention). Domain 2: Risk of bias due to deviations from the intended interventions (effect of adhering to intervention). Domain 3: Risk of bias due to missing outcome data. Domain 4: Risk of bias in measurement of the outcome. Domain 5: Risk of bias in selection of the reported result.

**Table 5** is a summary of the quality assessment of the cohort studies using the CASP checklist. All seven cohort studies (30–33, 35, 37, 40) scored well for questions 1, 4, 7, 8 and 9, thus displaying focus and sufficient measurement of outcomes and appropriate follow-up (**Table 5**). All studies were judged to have recruited the cohorts in an acceptable way except for one (31), due to the uneven group sizes and other significant differences in baseline characteristics. The measurement of exposure was deemed unclear in three studies. Walter et al. (37) failed to mention confounding factors in their study and Karyampudi et al. (35) were unable to take into account confounding factors as their small sample size precluded them from carrying out a multivariate logistic regression analysis. The level of precision of six of the cohort studies was deemed to be unclear due to the absence of confidence intervals. Overall, the cohort studies were regarded as having no or unclear implications for clinical practice due to small sample sizes, insignificant results and lack of conformity with other studies.

**Table 5:** Critical Appraisal Skills Programme Cohort Study checklist.

Authors	1	2	3	4	5	6	7	8	9	10	11	12	13	14
Bogazzi et al, 2010 (40)														
Eschmann et al, 2006 (30)														
Karyampudi et al, 2014 (35)														
Körber et al, 2001 (31)														
Santos et al, 2004 (32)														
Walter et al, 2009 (37)														
Zantut-Wittman et al, 2005 (33)														

Red indicates high risk of bias, yellow indicates unclear risk of bias and green indicates low risk of bias. Questions in checklist are: 1. Did the study address a clearly focused research question? 2. Was the cohort recruited in an acceptable way? 3. Was the exposure accurately measured to minimize bias? 4. Was the outcome accurately measured to minimize bias? 5. Have the authors identified all important confounding factors? 6. Have they taken account of the confounding factors in the design and/or analysis? 7. Was the follow up of subjects complete enough? 8. Was the follow up of subjects long enough? 9. What are the results of this study? 10. How precise are the results? 11. Do you believe the results? 12. Can the results be applied to the local population? 13. Do the results of this study fit with other available evidence? 14. What are the implications of this study for practice?

Although the design of the case-control study (34) seemed appropriate, there were some uncertainties regarding the selection of the cases and controls due to lack of information. Furthermore, due to the small sample size, it would seem inappropriate to generalize the findings of this study to the rest of the population. A summary of this quality assessment can be seen in **Table 6**.

**Table 6:** Critical Appraisal Skills Programme Case-Control Study checklist.

Authors	1	2	3	4	5	6	7	8	9	10	11	12
Oszukowska et al, 2010 (34)												

Red indicates high risk of bias, yellow indicates unclear risk of bias and green indicates low risk of bias. Questions in checklist are: 1. Did the study address a clearly focused research question? 2. Did the authors use an appropriate method to answer their question? 3. Were the cases recruited in an acceptable way? 4. Were the controls selected in an acceptable way? 5. Was the exposure accurately measured to minimize bias? 6. Aside from the experimental intervention, were the groups treated equally? 7. Have the authors taken account of the potential confounding factors in the design and/or in their analysis? 8. How large was the treatment effect? 9. How precise was the estimate of the treatment effect? 10. Do you believe the results? 11. Can the results be applied to the local population? 12. Do the results of this study fit with other available evidence?

### Hyperthyroidism

While carbimazole reduced treatment success (defined as euthyroidism or hypothyroidism) in hyperthyroid patients overall, Walter et al. (36, 37) found that the impact was more pronounced in patients with Graves’ disease than in patients with toxic nodular hyperthyroidism. Körber et al. (31) found that there was a significant reduction in treatment success between patients with toxic nodular goitre who took antithyroid drugs during and at least 14 days after RAI administration (discontinued one week before study endpoint measurements) and those who discontinued at least 14 days prior to RAI administration, but observed a non-significant reduction in patients with Graves’ disease. Karyampudi et al. (35) found no significant effects of carbimazole on cure rates. However, they report that in patients who were pre-treated with carbimazole, those who took it for a shorter period were more likely to yield treatment failure (persistent hyperthyroidism) (35). Eschmann et al. (30) found that significantly fewer patients achieved treatment success (euthyroidism or hypothyroidism) if they continued to take antithyroid drugs (carbimazole, methimazole or propylthiouracil) compared to the group who discontinued these drugs 3-7 days before RAI.

Two studies considered the effect of ‘block and replace’ therapy. Bonnema et al. (28) found that a significant proportion of patients who continued ‘block and replace therapy’ during RAI administration were not cured in comparison to the control. This effect was observed in patients with toxic nodular hyperthyroidism but not in patients with Graves’ disease. Counter to this, Andrade et al. (26) found no significant effects of methimazole pre-treatment on treatment outcomes and attribute this to the withdrawal of methimazole 4 days before RAI administration in conjunction with the high RAI dose use. Similarly, two studies found no significant differences in outcomes when patients continued to take methimazole during RAI administration (27, 29). Zantut-Wittman et al. (33) found no significant relationship between the use of methimazole or propylthiouracil but did find that overall, antithyroid drug withdrawal 2-30 days before RAI administration was associated with treatment success in comparison to continuation. Santos et al. (32) reported propylthiouracil being more associated with treatment failure than methimazole.

In their case-control study, Oszukowska et al. (34) found that RAI administration was significantly less effective in patients who were pre-treated with thiamazole. The effect of prednisone was investigated in patients who had Graves’ orbitopathy in the same study and it was found that patients who had taken prednisone before RAI administration achieved a higher cure rate than those in the control group, who did not have Graves’ orbitopathy and were given RAI only (34). Although Oszukowska et al. (34) had no significant findings regarding lithium carbonate and RAI treatment outcomes, Płazińska et al. (41) found that patients who received lithium carbonate 6 days prior to and 3 days after RAI administration were more likely to achieve euthyroidism than those who did not receive lithium carbonate pre-treatment. However, these findings were only significant in the first year following RAI administration (41).

Out of the three studies analyzing the effects of lithium on RAI outcomes (38–40), two studies (38, 39) reported no significant effects on cure rate (defined as stable euthyroidism (38) or permanent hypothyroidism (39)), though Bogazzi et al. (39) note that hyperthyroidism was controlled faster in the two groups who either received lithium five days before RAI administration (for 19 days) or on the day of RAI administration (for six days) compared to the group did not receive lithium. Bogazzi et al. (40) defined cure as permanent hypothyroidism or stable euthyroidism. They (40) found cure rates were higher in the group who had received lithium five days before and seven days after RAI administration than in the group who did not receive lithium (40).

### Thyroid cancer

Wen et al. (25) found that pre-treatment with glycididazole sodium increases complete response rate in patients with differentiated thyroid cancer but has no significant effect on effective response rate. They also found Tg concentrations (unstimulated) were significantly lower in patients who had taken glycididazole sodium than those who had not at 12 weeks, although this difference was not observed at 24 weeks (25), suggesting this could be a transient effect. Tg was used as a surrogate marker in this study, and long-term outcomes including recurrence were not considered. The effects of other background medications were not considered in thyroid cancer patients.

### Meta-analysis

**Figure 2:**
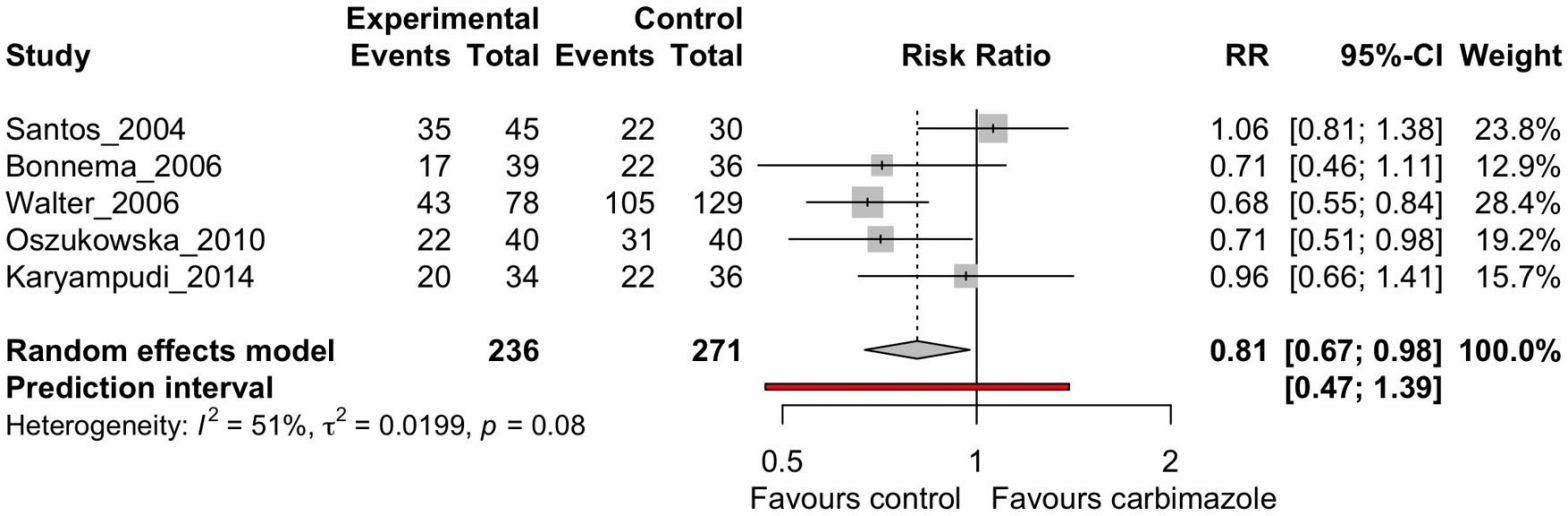
Random effects meta-analysis of carbimazole/methimazole studies that considered cure, defined as euthyroidism or hypothyroidism, after radioactive iodine.

**Figure 3:**
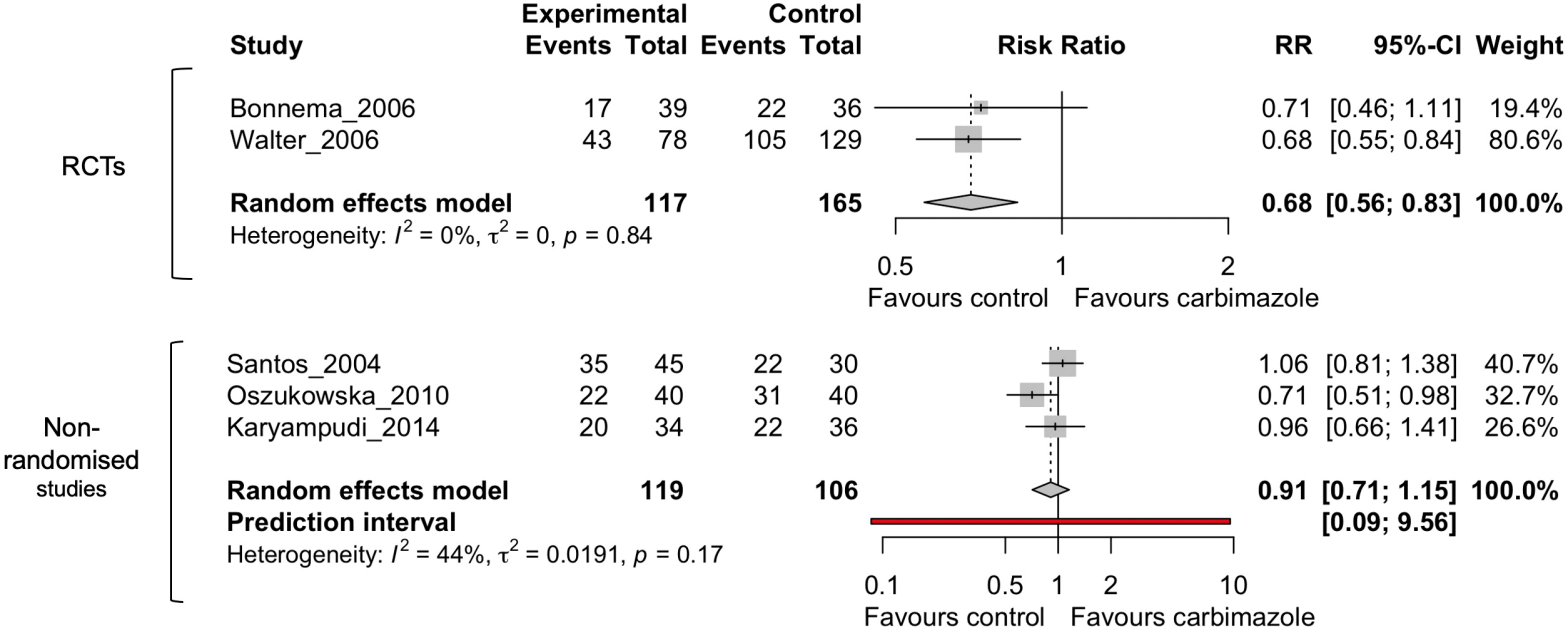
Random effects meta-analysis of carbimazole/methimazole studies divided into randomized control trials (RCTs) and non-randomized studies that considered cure after radioactive iodine.

Out of all the drugs assessed in these studies only methimazole and carbimazole (in hyperthyroidism) were investigated in multiple studies to consider meta-analysis (27, 32, 34–36). Results for other medications, including lithium, were too few to perform meta-analysis. No drugs were appropriate for meta-analysis in thyroid cancer due to the low number of studies. The I^2^ statistic was 51% for the 5 studies defined for this analysis (**Figure 2**), above what was defined in the original PROSPERO database registration (<50%). Therefore, a subset analysis was also performed dividing the studies into randomized-control trials and non-randomized studies (**Figure 3**).

The subset analysis dividing randomized and non-randomized studies (**Figure 3**) showed an I^2^ of 0% for the RCTs and 44% for the non-randomized studies, indicating the heterogeneity is between these two groups and in the non-randomized studies. However, as there were only two RCTs, additional clinical studies will be needed to improve meaningful conclusions from these comparisons.

The overall analysis (**Figure 2**) indicates there was moderate heterogeneity between the studies when all analyzed together (I^2^ = 51%, τ^2^ = 0.0199, p = 0.08). The random effects model did show favorable treatment for the patients who were not on the drug carbimazole or methimazole (RR = 0.81, p = 0.02), supporting the findings of the qualitative systematic review. The prediction interval did however span the RR of 1, which is consistent with the study heterogeneity findings. These findings of our meta-analysis demonstrate that patients with hyperthyroidism undergoing RAI therapy have worse cure rates if they are on antithyroid drugs such as carbimazole or methimazole.

## Discussion

### Hyperthyroidism

Of the 17 studies evaluated in this systematic review, 12 investigated the effect of antithyroid drugs (carbimazole, methimazole, thiamazole and propylthiouracil) on RAI therapy outcomes in hyperthyroid patients. This finding in itself demonstrates that the literature disproportionately represents thyroid related medication only, in terms of impact on RAI therapy. Our systematic review findings are largely consistent with those by Walter et al. (16) that antithyroid drugs have a negative impact on hyperthyroid patients’ post-RAI therapy outcomes, seen as a reduction in success rate of RAI treatment.

Results from the five studies (30, 31, 35–37) involving carbimazole imply that the drug may be having negative effects on the success of RAI treatment in hyperthyroid patients, but the effect on toxic nodular goitre patients versus Graves’ disease patients seems to be inconsistent. It is interesting to note however, that univariate analysis by Karyampudi et al. (35) demonstrated that patients who took carbimazole for a shorter of period of time prior to RAI were more likely to remain hyperthyroid, highlighting a further area of research in this area.

Prednisone is a commonly prescribed corticosteroid with high glucocorticoid activity. Prednisone correlated with an increase in RAI treatment success, however it was only studied in one paper (34) hence no robust conclusions can be made. No significant effects of lithium carbonate were found in our review.

Studies of the effect of lithium were limited hence it would not be appropriate to form conclusions regarding this drug. However, lithium is a commonly prescribed drug in thyroid disease therefore, necessitating further information about its actions.

The meta-analysis in this study looked at cure rate, defined as euthyroidism or hypothyroidism, after radioactive iodine in patients who were taking carbimazole/methimazole versus control, which was limited to five studies. The antithyroid medication studies were the only studies that were numerate enough to perform a quantitative analysis. The I^2^ value was 51%, demonstrating moderate heterogeneity to the studies analyzed. The meta-analysis demonstrates an improved rate of cure when patients are not taking antithyroid drugs, which is in keeping with the qualitative systematic review findings.

Papers were heavily focused on thyroid-related medications, which demonstrates the paucity of data on a patients’ background medications and how these might affect RAI function.

### Thyroid cancer

Enhanced understanding of the factors which impact thyroid cancer treatment success is required to address the ~45,000 lives lost worldwide to thyroid cancer per annum, which is estimated to rise to 74,733 by 2040 (42). In a screen of FDA-approved drugs, we recently demonstrated that common medications such as digoxin and pioglitazone gave negative values for the surrogate marker indicating reduced intracellular iodide, whereas clotrimazole and mifepristone gave positive values for the marker denoting increased intracellular iodide (21). These findings further underscore the need to investigate whether common medications taken by patients given RAI therapy impact on patient prognosis.

In the systematic review there was only one study specific to thyroid cancer (25), in which the effect of glycididazole sodium was explored. Glycididazole sodium is used as a radiosensitizing agent for radiotherapy in hypoxic tumors, but has not been extensively investigated in radioactive iodine therapy. Given that Wen et al. (25) reported increased complete response rates with glycididazole sodium, we suggest further trials should be performed to gain clarity on the effect the drug in thyroid cancer.

## Limitations

One major drawback of this review is the quality of the studies evaluated. A majority of the RCTs were judged to be of high concern for risk of bias and all of the cohort studies were found to have either no or unclear implications for practice. This stems from the rather small sample sizes. In addition to this, only 17 studies were eligible for review, with only one pertaining to thyroid cancer patients. While it appears the effects of some drugs are clear, this may not be a true reflection of outcomes due to these drawbacks. We excluded non-English language studies which limited the studies we reviewed. Another limitation is that although we were able to assess results of each study, the outcome measurements differed between them.

The meta-analysis in this study was limited by the low number of studies included, and also by the study heterogeneity. Only the carbimazole/methimazole studies had enough studies with comparable outcomes (cure post-radioiodine treatment) to encourage meta-analysis. On registration of the systematic review in the PROSPERO database, performing a meta-analysis was limited to over five studies and a I^2^ heterogeneity value of < 50%. An I^2^ value of 51% was discovered in this review, but the meta-analysis has been included nonetheless.

## Conclusions

Antithyroid drugs including carbimazole, methimazole, thiamazole and propylthiouracil have a negative impact on RAI outcomes in patients with hyperthyroidism. It seems that lithium, lithium carbonate and prednisone may have a positive impact on RAI outcomes in patients with hyperthyroidism, albeit this remains an area where further work is needed. In thyroid cancer patients, the effects of glycididazole sodium remain unclear. Our systematic review strengthens previous work, however this field remains under researched especially for medications unrelated to thyroid disease, with very few papers on non-thyroid medications published. Larger randomized-control trials with more uniform standards (e.g. definitions and measurements of thyroid status, length of follow up periods, outcome measurements) and larger scale observational studies investigating drugs commonly taken by thyroid cancer and hyperthyroid patients are urgently needed to inform clinical practice.

## Data availability statement

The original contributions presented in the study are included in the article/**Supplementary Material**. Further inquiries can be directed to the corresponding author.

## Author contributions

Authors contributed to the systematic review as reported: Study design conception, HN and JM, data collection and analysis, RZ, JL, and HN, manuscript writing, RZ, MR, HN, and CM, manuscript editing, RZ, JL, JM, MR, KBr, NS, KBo, CM, and HN, supervision, HN and CM. All authors contributed to the article and approved the submitted version.
