# Supplementary material for: The potential interaction between medical treatment and radioiodine treatment success: A systematic review
Source: Front Endocrinol (Lausanne). 2023 Jan 4;13:1061555. doi: 10.3389/fendo.2022.1061555 (PMC9845773; doi:10.3389/fendo.2022.1061555)
Supplement: Supplementary file 1 [file Table_1.docx]

Supplementary Material

# Supplementary Tables

| Search term | Result |
| --- | --- |
| 1. (hyperthyroidism).ti,ab | 25301 |
| 1. ("overactive thyroid").ti,ab | 43 |
| 1. (Thyroid ADJ1 (Cancer OR neoplasms)).ti,ab | 34997 |
| 1. ("papillary carcinoma").ti,ab | 7817 |
| 1. ("follicular carcinoma").ti,ab | 2813 |
| 1. ((Medullary OR anaplastic) ADJ1 "thyroid carcinoma").ti,ab | 6795 |
| 1. (34 OR 35 OR 36 OR 37 OR 38 OR 39) | 71832 |
| 1. ("radioactive iodine").ti,ab | 6635 |
| 1. (Radioiodine).ti,ab | 11212 |
| 1. (I-131).ti,ab | 4072 |
| 1. (41 OR 42 OR 43) | 19514 |
| 1. (Amiodarone).ti,ab | 14846 |
| 1. (Alendronate).ti,ab | 7608 |
| 1. Clindamycin | 0 |
| 1. (Escitalopram).ti,ab | 4777 |
| 1. (Mebeverine).ti,ab | 249 |
| 1. ("Enalapril maleate").ti,ab | 552 |
| 1. (Diltiazem).ti,ab | 11107 |
| 1. (Amitryptiline).ti,ab | 295 |
| 1. (Exemestane).ti,ab | 2614 |
| 1. Raloxifene | 0 |
| 1. (Spironolactone).ti,ab | 9218 |
| 1. (Alfacalcidol).ti,ab | 1057 |
| 1. (Digoxin).ti,ab | 15746 |
| 1. (Celecoxib).ti,ab | 9471 |
| 1. ("Cyproterone acetate").ti,ab | 2994 |
| 1. (45 OR 46 OR 47 OR 48 OR 49 OR 50 OR 51 OR 52 OR 53 OR 54 OR 55 OR 56 OR 57 OR 58 OR 59) | 97057 |
| 1. (Medication).ti,ab | 382575 |
| 1. (Medicine).ti,ab | 754180 |
| 1. (Drug).ti,ab | 1711872 |
| 1. (60 OR 61 OR 62 OR 63) | 2759604 |
| 1. (40 AND 44 AND 64) | 1487 |
| 1. (refractory).ti,ab | 224819 |
| 1. (40 AND 44) NOT 66 | 9220 |
| 1. (64 AND 67) | 1276 |

Supplementary Table 1. Search strategy on EMBASE for records published between 2001 and 2021. ti = title; ab = abstract.
